# Circular RNAs: pivotal role in the leukemogenesis and novel indicators for the diagnosis and prognosis of acute myeloid leukemia

**DOI:** 10.3389/fonc.2023.1149187

**Published:** 2023-04-14

**Authors:** Atefe Rahmati, Alireza Mafi, Firooze Soleymani, Zahra Babaei Aghdam, Niloufar Masihipour, Behrooz Ghezelbash, Reza Asemi, Michael Aschner, Omid Vakili, Mina Homayoonfal, Zatollah Asemi, Mehran Sharifi, Abbas Azadi, Hamed Mirzaei, Esmat Aghadavod

**Affiliations:** ^1^ Department of Hematology and Blood Banking, Faculty of Medicine, Mashhad University of Medical Sciences, Mashhad, Iran; ^2^ Department of Basic Sciences, Faculty of Medicine, Neyshabur University of Medical Sciences, Neyshabur, Iran; ^3^ Department of Clinical Biochemistry, School of Pharmacy and Pharmaceutical Sciences, Isfahan University of Medical Sciences, Isfahan, Iran; ^4^ Department of Medical Biotechnology and Nanotechnology, Faculty of Medicine, Mashhad University of Medical Sciences, Mashhad, Iran; ^5^ Imaging Sciences Research Group, Tabriz University of Medical Sciences, Tabriz, Iran; ^6^ Department of Medicine, Lorestan University of Medical Science, Lorestan, Iran; ^7^ Department of Immunology, School of Medicine, Isfahan University of Medical Sciences, Isfahan, Iran; ^8^ Department of Internal Medicine, School of Medicine, Cancer Prevention Research Center, Seyyed Al-Shohada Hospital, Isfahan University of Medical Sciences, Isfahan, Iran; ^9^ Department of Molecular Pharmacology, Albert Einstein College of Medicine, Bronx, NY, United States; ^10^ Research Center for Biochemistry and Nutrition in Metabolic Diseases, Kashan University of Medical Sciences, Kashan, Iran; ^11^ Department of Internal Medicine, Lorestan University of Medical Sciences, Khorramabad, Iran; ^12^ Department of Clinical Biochemistry, School of Medicine, Kashan University of Medical Sciences, Kashan, Iran

**Keywords:** circular RNA (circRNA), microRNA, acute myeloid leukemia, biomarker, targeted therapy

## Abstract

Acute myeloid leukemia (AML) is an aggressive hematological malignancy and affected patients have poor overall survival (OS) rates. Circular RNAs (circRNAs) are a novel class of non-coding RNAs (ncRNAs) with a unique loop structure. In recent years, with the development of high-throughput RNA sequencing, many circRNAs have been identified exhibiting either up-regulation or down-regulation in AML patients compared with healthy controls. Recent studies have reported that circRNAs regulate leukemia cell proliferation, stemness, and apoptosis, both positively and negatively. Additionally, circRNAs could be promising biomarkers and therapeutic targets in AML. In this study, we present a comprehensive review of the regulatory roles and potentials of a number of dysregulated circRNAs in AML.

## 1 Introduction

Acute myeloid leukemia (AML) is a clonal, aggressive malignancy of hematopoietic tissues that is characterized by the block of myeloid differentiation and expansion of immature myeloid progenitors in the bone marrow (BM) (1, 2). Various epigenetic and genetic abnormalities can arrest hematopoietic cell differentiation and maturation steps, leading to clonal expansion within the BM and blood (3, 4). This leads to a range of fetal clinical problems, including infections, bleeding events, and organ damage. Owing to advances in cytogenetics, molecular biology, and next-generation sequencing (NGS), the classification of AML has shifted from a morphology-based classification to a classification algorithm that prioritize genetic abnormalities to establish diagnosis and prognosis (5).

Genomic instability plays a pathologic role in AML. It ranges from mutations in proliferation/survival mechanisms and differentiation/apoptosis pathways to chromosomal structure and numerical abnormalities, frequently found in leukemia cells (6). Of the known genetic alterations in leukemia, chromosomal translocations leading to the formation of the oncogenic fusion protein or oncogene activation by a new enhancer or promoter elements are among the most common rearrangements (7). Despite a long history of leukemia research and therapy improvements, AML patients have poor survival rates. Relapse with the more aggressive and resistant disease is common, even in patients who achieved complete remission (CR) after standard induction chemotherapy and/or hematopoietic stem cell transplantation (8, 9).

AML is a heterogeneous disease, and the extent to which cytogenetic and molecular characteristics define severity and influence therapeutic decisions is a rapidly evolving area of investigation.

The relevance of genomic characterization has also been reflected in the European LeukemiaNET (ELN) risk classification. In 2017 edition of the ELN, the risk stratification based on cytogenetic analysis failed to accurately predict the disease course in some cases, because either patients that were categorized in the intermediate-risk group show highly variable outcomes or had survival predictions deviating more than 20% from their ELN statum (10, 11).

Although the latest edition of this genetic-risk classification of AML seemed to accurately reflect the individual patient’s treatment outcomes in patients aged <60 years, in older patients, relapse rate, disease-free (DFS) and overall (OS) survival were not significantly different between intermediate and adverse groups (12). Most AML patients still lack actionable targets for successful targeting and long-term survival remains low (5-year OS 28.7%) (13). Thus, discovering new biomarkers for disease diagnosis, stratification of the prognosis system, and enhancing treatment decision-making has been one of the hotspots in recent years.

After years of believing that a large proportion of eukaryotic genes encodes no proteins, it has been realized that they constitute a majority of expressed RNAs compared to protein-coding transcripts and that small fractions of these genes have important essential regulatory roles (14, 15). Currently, non-coding RNAs (ncRNAs) can be classified by length (small ≤ 200 nt; long > 200 nt) or functionality [housekeeping RNAs such as ribosomal RNAs (rRNAs) and transfer RNAs (tRNAs)] as well as regulatory RNAs [such as microRNAs (miRNAs), small nuclear RNAs (snRNAs), piwi-interacting RNAs (piRNAs) and long non-coding RNAs (lncRNAs)] (16). Aberrant expression of ncRNAs is closely associated with many cancers, including AML. Likewise, the regulatory mechanisms to control their expression have been implicated in cancer diagnosis, prognosis, treatment, and cancer pathogenesis (17, 18).

CircRNAs are a type of single-stranded and non-polyadenylated class of ncRNAs with enclosed configurations. CircRNAs are commonly found in human cells and are more diverse and abundant than their linear counterparts. CircRNA expression is dynamically regulated and spatiotemporal-specific among cell types, tissues, and developmental stages, rendering them ideal diagnostic biomarkers for cancer. Moreover, circRNAs modulate the expression or function of their host genes or participate in the regulation of oncogenic signaling pathways, thereby contributing to cancer development (19, 20). Interestingly, during leukemogenesis, extracellular vesicles containing onco-circRNAs which released by leukemic cells could change the properties of BM microenvironment in favor of AML progression (21, 22).

A better understanding of different core players, such as circRNAs and their partners with oncogenic or tumor-suppressive characteristics, may help improving disease diagnosis, classification, and treatment strategies. This review aims to describe the regulatory roles of circRNAs in AML and sheds light on their potential use in the clinical setting, such as diagnostic/prognostic biomarkers and therapeutic targets.

## 2 An overview of circRNAs

### 2.1 From the discovery to biogenesis

CircRNAs are characterized by covalently closed continuous loop structures without free 5’ and 3’ ends; this circular structure makes them more stable against most RNA decay machinery than their linear counterparts (23). In 1976 circRNAs were identified with an electron microscopy-based study of a plant-based virus and belonged to a growing list of types of ncRNAs (24, 25). CircRNAs were first considered the outcome of transcriptional noise, and researchers ignored their importance during the following decades. Nevertheless, their essential function in gene regulation is now widely appreciated. The advances in high-throughput RNA sequencing (RNA-seq) and circRNA-specific bioinformatics algorithms accelerated the discovery of circRNAs (26). The maturation of mRNA is a well-orchestrated process divided into three phases: 5’- end capping, splicing, and 3’-end cleavage/polyadenylation (27). Back-splicing is the name of an uncommon alternative splicing responsible for generating circRNAs. These RNAs are in the reverse order of canonical splicing, and the whole process is catalyzed by either group I and II ribozymes or the spliceosomal machinery. They may produce through back-splicing of exons and introns, or both, to form exonic intronic and exonic-intronic circRNAs, respectively. As a result of a non-colinearly splicing event, a circRNA is generated *via* ligation of an upstream 3’ acceptor splice site to a downstream 5’ donor splice site (28).

**Figure 1:**
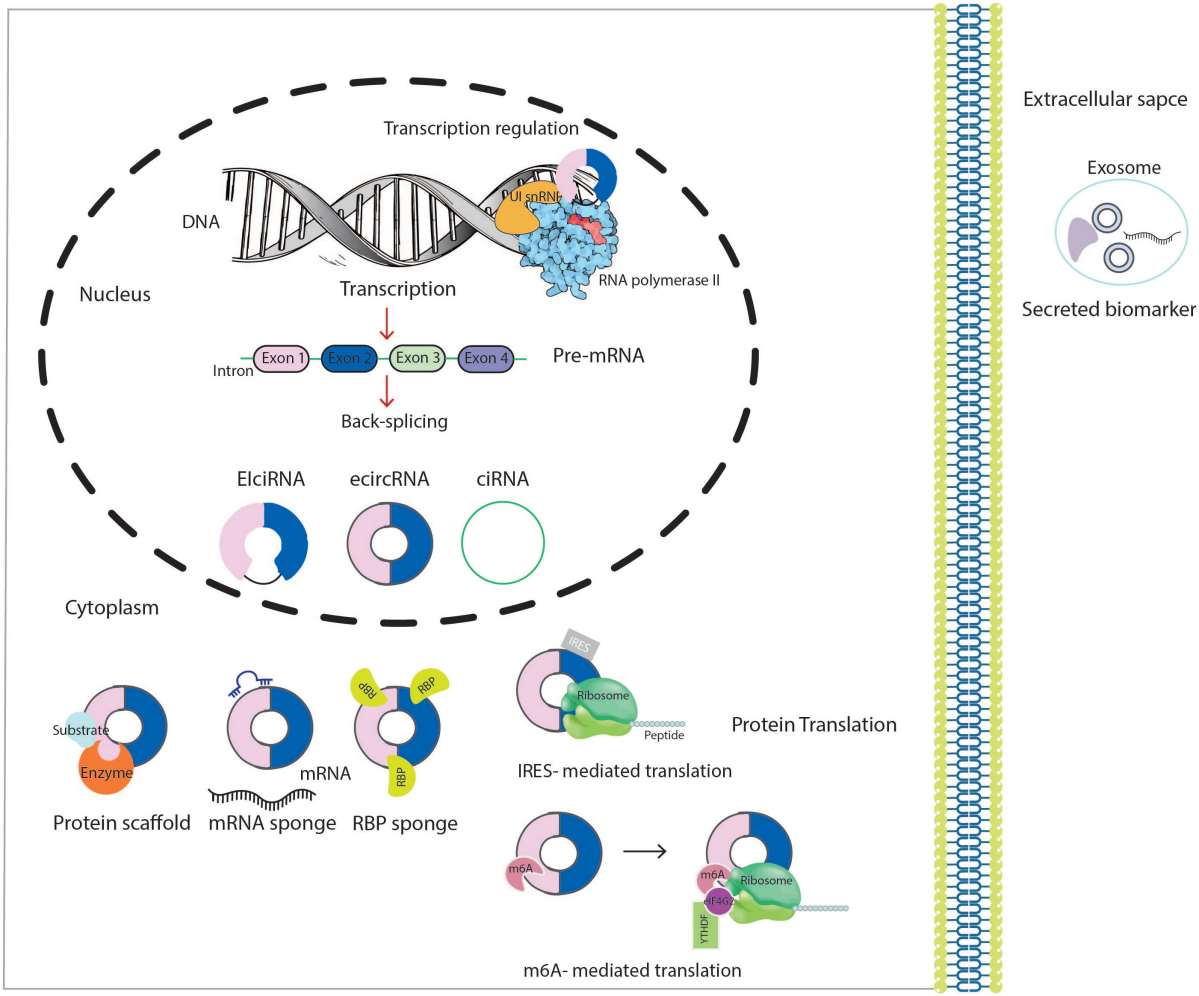
Schematic presentation of the biogenesis and biological functions of circRNAs. CircRNA, circular RNA; U1 snRNP, U1 small nuclear ribonucleoprotein; RBP, RNA-binding protein; IRES, internal ribosome entry sites; m6A, methyl adenosine; eIF4G2, eukaryotic translation initiation factor 4 gamma 2.

Many cis-acting and trans-acting elements could regulate circularization. It depends on the base-pairing ability between inverted complementary repeat elements (such as the repetitive *Alu* elements and non-repeated complementary sequence), primarily found in the upstream and downstream introns. In addition, RNA-binding proteins (RBPs) [such as heterogeneous nuclear ribonucleoprotein L (HNRNPL), protein quaking (QKI) and fused in sarcoma (FUS) RNA binding protein], spliceosome factors, cleavage factors (such as ESRP1) and RNA helicases (such as DHX9), which specifically recognize and bind to particular areas of the intron, are some of the trans-regulatory elements that influence configuration (29, 30). Two possible models of circRNAs biogenesis through back-splicing have been proposed: Lariat intermediate and direct back-splicing. The principal difference between these two models is in the first step of a circRNA generation. Additionally, three splicing models have been identified in the biogenesis of a circRNA: Lariat-driven cyclization, intron pairing driven circularization, and RBP-driven circularization. The exon-skipping model relies primarily on lariat-driven cyclization to splice pre-mRNA, whereas direct back-splicing relies upon RBP-mediated and intron-pairing cyclization. “Exon skipping” or “lariat intermediate” model suggests that the processes of a circRNA biogenesis begin with canonical splicing for a linear RNA with skipped exons and a long intron lariat containing exons and introns that are later back spliced in order to form a circRNA. Alternatively, in “direct back splicing,” a circRNA is generated by back splicing circRNA together with an intermediate containing both introns and exons, which can then be further processed to generate a linear RNA or destroyed (31, 32). CircRNAs have been divided into three main types according to their constituent parts and the location from which they arise in the genome including exonic circRNAs (ecircRNAs), exonic-intronic circRNAs (EIciRNAs) and circular intronic RNAs (ciRNAs) (**Figure 1**) (30, 33).

### 2.2 Molecular functions of circRNAs

CircRNAs modulate various physiological and pathological processes, such as proliferation, differentiation, migration, invasion and apoptosis to their role in drug resistance (34, 35). CircRNAs primarily function as regulatory ncRNAs at both transcriptional and post-transcriptional levels. CircRNAs interact directly with the RNA polymerase II (RNA Pol-II) machinery to stimulate the parental gene transcription (20, 36). For instance, EIciRNAs interact with U1 small nuclear ribonucleoproteins (U1 snRNP) and RNA Pol-II, thus regulating the promoter region of host gene transcription (37). Among its biological functions, circRNAs predominantly function as miRNA sponges. Accordingly, complementary circRNAs bind to miRNAs sequence and consequently inhibit their activity, upregulating or downregulating miRNA target gene expression. For example, in AML, circ-DLEU2 can act as a competing endogenous RNA (ceRNA) to bind directly to miR-496, thus stimulating cell proliferation and protecting target mRNAs from miRNA-dependent degradation (38). Moreover, a single circRNA may harbor several binding sites for multiple miRNAs, such as circ-NPM1 can suppress the activity of miR-345-5p, miR-765, miR-495-3p, miR-665, miR-193b-5p, and miR-124-5p under certain conditions (39, 40). However, not all circRNAs have miRNA binding sites and suppress miRNA expression. Rather than acting as miRNA sponges, circRNAs also serve as decoys by sequestering the proteins and RBPs, influencing the protein’s expression and function (20). For example, circHuR interacts with CCHC-type zinc finger nucleic acid-binding protein (CNBP) and blocks its binding to the HuR promoter, leading to reduced HuR expression to further inhibit gastric cancer progression (41). Some circRNAs may serve as protein scaffolds favoring the colocalization of enzymes and their substrates to enhance the reaction kinetics (34). A good example would be circ_Foxo3 which repressed cell cycle transition trough forming a ternary complex with cell cycle proteins cyclin-dependent kinase 2 (CDK2) and cyclin-dependent kinase inhibitor 1 (or p21). Acting as a scaffold and inhibiting the interactions between CDK2 and cyclin A and E end up blocking cell cycle progression (42).

CircRNA also work as protein recruiters in particular loci or subcellular compartments. For example, hsa_circ_0121582 was found to inhibit leukemia cell proliferation by recruiting DNA demethylase TET1 to the promoter region of GSK3β (43). Although circRNAs had been considered untranslatable for a long time, because of the lack of a 5’m7 G Cap and 3’ poly (A) tail, in the past few years, more and more circRNAs have been revealed to encode regulatory peptides and proteins. There are two translation initiation mechanisms of RNAs: cap-dependent and cap-independent. CircRNAs could undergo cap-independent translation mediated in an internal ribosome entry site (IRES) element-dependent manner. During this process, translation is initiated by directly binding 40S to IRES elements **(** **Figure 1** **)** (44).

## 3 The roles of circRNAs in leukemia

CircRNAs have been expressed in range of blood cell populations, with cell- and differentiation stage-specific expression, rendering such circRNAs as potential hematopoietic regulators (45, 46). One of the earliest studies to describe circRNAs in hematopoiesis was published in 1998 by Caldas et al. (47) in which they introduced splicing of the MLL gene, a frequently rearranged gene in a variety of hematological malignancies, which is involved in a complex process that results in the formation of abnormal transcripts with different genomic orientation, scrambled transcripts, and circRNAs in both normal and leukemic cells. Research on hematopoiesis-related circRNAs has grown rapidly since NGS emerged, and the catalog of these circRNAs has dramatically expanded (48). For example, it is known that circ-BACH1 (exons 2-4) is mainly expressed in hematopoietic stem cells (HSCs) and multipotent progenitors (MPPs), whereas circ-FNDC3B (exon 5-6) is broadly expressed in natural killer (NK) cells, circ-ELK4 (exon 4-3), circ-SLFN12L (exon 2-3) and circ-MYBL1 (exon 12-7) are preferentially expressed in T cells and NK cells, circ-AKT3 and circ-CCDC91 were found to be expressed across lymphoid cells (49).

CircRNAs actively participate in key physiological and cellular processes such as self-renewal, proliferation, and apoptosis of these hematopoietic compartments (50). In addition to their role in physiological conditions, there is a relationship between altered circRNAs expression and malignant hematopoiesis. Increasing evidence suggests that circRNAs may involve leukemic transformation mainly through regulation of the gene expression by sponging or decoying specific miRNAs (45). miRNAs are critical regulators in posttranscriptional regulation of AML and activation of downstream signaling pathways associated with migration, differentiation, and proliferation. Therefore, a dysregulated expression of miRNA level disturbs normal hematopoiesis, and leukemia may emerge (51). In addition, circRNAs can also induce leukemia transformation by direct RBP sponging or indirect circRNA-miRNA-RBP interactions since RBPs are also involved in biological processes like cell cycle progression (52). Molecular biology and gene expression studies using various strategies including RNA-seq, microarray, PCR array, northern blot, and *in situ* hybridization techniques have begun to map out the expression profiles of circRNAs in hematological malignancies, especially AML (53). Specific circRNA-miRNA-mRNA axes have been shown to regulate leukemia-related processes and serve as diagnostic, prognostic, and therapeutic biomarkers (54).

### 3.1 Dysregulated circRNAs in AML

**Figure 2:**
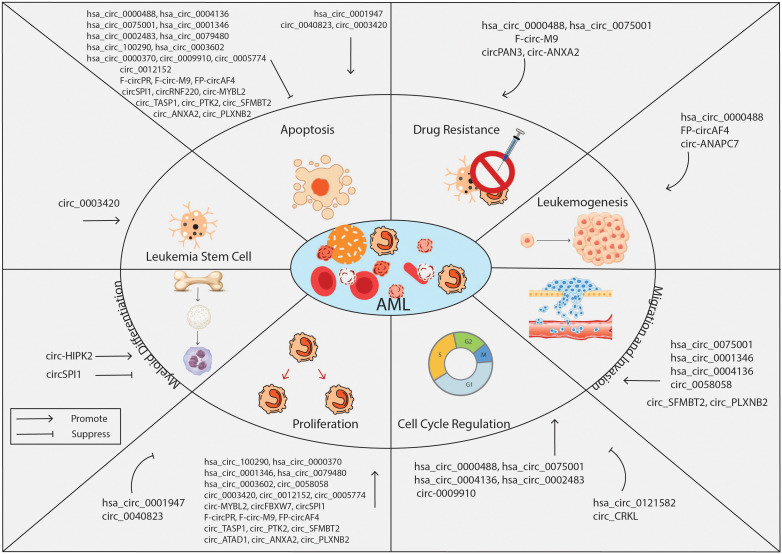
Overview of the biological roles of circRNAs in AML. CircRNAs are known to function either as oncogenes or tumor suppressor genes, regulating key cell processes of leukemia cells such as proliferation, apoptosis, cell cycle, stemness, drug resistance, migration, and invasion. CircRNA, circular RNA; AML, acute myeloid leukemia.

Several studies reported the aberrant expression of circRNAs in leukemia biogenesis, maintenance, and progression. CircRNAs regulate gene expression and contribute to the pathological events involved in AML development, including cell transformation, myeloid differentiation, proliferation, cell cycle progression, and apoptosis. Recent studies revealed that circRNAs can function as oncogenes or tumor-suppressors genes. The oncogenic role of these circRNAs is demonstrated by the repression of tumor suppressor genes which leads to leukemia initiation and progression. In contrast, circRNAs with tumor suppressive role decrease the translation of oncogenes, leading to an inhibition of tumorigenesis. Below, we describe the most studies circRNAs in AML and the mechanisms by which they exert these functions. The role of circRNAs in AML is summarized in **Table 1** and **Figure 2**.

**Table 1:** CircRNAs involved in the pathogenesis of AML.

CircRNA	Target miRNA (s)	Expression	Type of model	Type of specimen(s) or cell line(s)	Corresponding signaling/protein	Function role	Ref.
hsa_circ_0000488 (circRNA-DLEU2)	miR-496	Up-regulated	Human, animal, *in vitro*	BM sample, C57BL/6 Mice, AML cell lines (MOLM-13, HL-60, MV-4-11)	PRKACB	Induction of proliferation, tumor formation inhibition of apoptosis	(38)
hsa_circ_100290	miR-203	Up-regulated	Human, *in vitro*	BM sample, AML cell lines (MV-4-11, Kasumi-1, HL-60 and AML2)	cyclin D1, CDK4, Bcl-2, Rab10	Induction of proliferation inhibition of apoptosis	(55)
circ-MYBL2		Up-regulated	Animal, *in vitro*	NOD-SCID Mice, FLT3-ITD+ AML cell lines (MV4-11, MOLM-13), FLT3-ITD- AML cells (THP-1, HL60, NB4, ML-2, U937, K562), 293t cells	FLT3-ITD PTBP1	Induction of proliferation and colony formation, inhibition of apoptosis	(56)
circFBXW7		Down-regulated	Human	Blood and BM samples		Inhibition of proliferation	(57)
hsa_circ_ 0000370	miR-1299, (miR-370-3p, miR- 502-5p, miR-640, miR-1281)	Up-regulated	Human, *in vitro*	Blood sample, AML cell lines (HL-60, THP-1, U937, K-562, MV4-11, SEM-K2, Molm14, HB19;16)	S100A7A	Induction of cell viability and colony formation, inhibition of apoptosis	(58)
hsa_circ_0075001 (circ-NPM1)	miR-765, miR-495-3p, miR-665, miR-193b-5p, miR-124-5p	Up-regulated	Human, *in vitro*	BM sample, AML cell lines (Oci-AML5, NB4, K-562, ME-1, Oci-AML3, KASUMI-1, MV4-11)	TLR, CD14	Downregulation of TLR signaling pathway	(39)
hsa_circ_0075001 (circ-NPM1)	miR-345-5p	Up-regulated	Human, *in vitro*	Blood sample, AML cell lines (THP-1, HL-60)	FZD5	Induction of proliferation, cell cycle progression, colony formation, migration, and invasion, inhibition of apoptosis	(40)
f-circPR		Up-regulated	Human, animal, *in vitro*	BM sample, Mice, AML cell lines (THP-1, K562, U937, HL-60, Kasumi, NB4)	PI3K, MAPK	Induction of proliferation, inhibition of apoptosis	(59)
f-circ-M9		Up-regulated	Human, animal, *in vitro*	BM sample, Mice, AML cell lines (THP-1, K562, U937, HL-60, Kasumi, NB4)	PI3K, MAPK	Induction of proliferation, colony formation capacity, LSC viability, and therapeutic resistance, inhibition of apoptosis	(59)
fp- circAF4	miR-128-3p	Up-regulated	Human, animal, *in vitro*	BM sample, NOD-SCID Mice, AML cell lines (RS4;11, SUPB15, MV4-11)	MLL-AF4	Induction of proliferation and leukemogenesis, inhibition of apoptosis	(60)
circSPI1	miR-382-5p, miR-1307-3p, miR-767-5p	Up-regulated	Human, *in vitro*	Blood sample, AML cell lines (THP-1, NB4, HL60), HEK-293T	elF4AIII	Induction of proliferation, myeloid differentiation, inhibition of apoptosis	(61)
hsa-circ-0001346	miR-1224-5p	Up-regulated	Human, *in vitro*	Blood sample, AML cell lines (HL-60, Kasumi-1)	C-myc, Caspase3/7, Tenascin-C	Induction of proliferation, colony formation, migration, and invasion, inhibition of apoptosis	(62)
hsa_circ_0004136	miR-29a, miR-196a, miR-142-3p	Up-regulated	Human, *in vitro*	BM sample, AML cell line (Oci-AML3)		Induction of proliferation	(63)
hsa_circ_0004136	miR-570-3p	Up-regulated	Human, *in vitro*	Blood sample, AML cell lines (HL60) K562 and immortalized HS-5	TSPAN3	Induction of proliferation, migration, invasion, cell viability, and cell cycle progression, inhibition of apoptosis	(64)
circ_0005774	miR-192-5p	Up-regulated	Human, *in vitro*	Blood sample, AML cell lines (HL-60, NB4)	ULK1	Induction of proliferation, cell viability, and cell cycle progression, inhibition of apoptosis	(65)
circRNF220	miR-30a	Up-regulated	Human, *in vitro*	BM and blood samples, AML cell lines (HL-60, THP-1, K562)	MYSM1, IER2	Induction of proliferation, inhibition of apoptosis	(66)
circ-ANAPC7	miR-181a-5p, miR-181b-5p, miR-181d-5p, miR-338-3p, miR-562b-5p	Up-regulated	Human	BM sample		AML tumorigenesis	(67)
hsa_circ_0002483	miR-758-3p	Up-regulated	Human, *in vitro*	BM sample, AML cell lines (HL-60, AML-193)	MYC	Induction of proliferation, cell cycle transition, cell viability	(68)
hsa_circ_0079480	miR-654-3p	Up-regulated	Human, *in vitro*	BM sample, AML cell lines (MOLM-13, AML-193)	HDGF	Induction of proliferation, cell viability	(69)
circ-0009910	miR-20a-5p	Up-regulated	Human, *in vitro*	BM sample, AML cell lines (Mono-Mac-6, KG-1, AML2, AML5), human cell line 1D3		Induction of proliferation	(70)
circ-0009910	miR-5193-3p	Up-regulated	Human, *in vitro*	BM sample, AML cell line (HL-60, MOLM-13)	GRB10	Induction of proliferation and cell cycle, inhibition of apoptosis	(71)
circ-0009910	miR491-5p	Up-regulated	Human, *in vitro*	BM sample, AML cell line (HL-60)	B4GALT5, PI3K/AKT	Induction of proliferation and sphere formation, inhibition of apoptosis	(72)
hsa_circ_0121582	miR-224/	Down-regulated	Human, animal, *in vitro*	BM sample, Mice, AML cell lines (BDCM, HL-60, Kasumi-3, KG-1)	GSK3 β, Wnt/β-catenin	Inhibition of cell proliferation	(43)
hsa-circ_0001947	hsa-miR-329-5p, hsa-miR-10b-3p, hsa-miR-488-3p	Down-regulated	Human, animal, *in vitro*	BM sample, Mice, AML cell lines (THP-1)	CREBRF, KLHL15, KRT12, INTS9	Inhibition of cell proliferation	(73)
circ_0040823	miR-196a, miR-196b	Down-regulated	Human, *in vitro*	BM sample, AML cell lines (KG-1a, KG-1, Kasumi-1)	P27	Inhibition of proliferation, induction of apoptosis	(74)
circ_CRKL	miR-196a-5p, miR-196b-5p	Down-regulated	Human, *in vitro*	BM sample, AML cell lines (KG-1a, Kasumi-1)	P27	Suppression of the cell proliferation, cell cycle transition, colony-forming capacity	(75)
circ_0003420		Down-regulated	Human, animal, *in vitro*	BM sample, NOD/SCID mice, AML cell lines (KG-1a, THP-I, KASUMI-1)	IGF2BP1	Suppression of the characteristics of LSCs	(76)
hsa_circ_0003602	miR-502-5p	Up-regulated		BM sample, AML cell lines (HL-60, K562, and THP-1)	IGFR1	Induction of proliferation, inhibition of apoptosis	(77)
circ_TASP1	miR-515-5p	Up-regulated	Human, *in vitro*	Blood sample, AML cell lines (HL60, KG-1, U937, THP-1)	HMGA2, Wnt/β-catenin	Induction of proliferation, inhibition of apoptosis	(78)
circ_PTK2	miR-330-5p	Up-regulated	Human, *in vitro*	BM sample, AML cell lines (U937, Kasumi-1, K562, HL-60)	FOXM1	Induction of proliferation, inhibition of apoptosis	(79)
circ_0058058	miR-4319	Up-regulated			EIF5A2	Induction of proliferation, migration, and invasion, inhibition of apoptosis	(80)
circ_SFMBT2	miR-582-3p	Up-regulated	Human, *in vitro*	Blood sample, AML cell lines (HL-60, U937, NB4, K562)	ZBTB20	Induction of proliferation, migration, invasion, and glycolysis, inhibition of apoptosis	(81)
circ-ATAD1	miR-34b	Up-regulated	Human, *in vitro*	BM sample, AML cell lines (Kasumi-3, Kasumi-6)		Induction of cell proliferation	(82)
circPAN3	miR-153-5p, miR-183-5p	Up-regulated	Human, *in vitro*	BM sample, AML cell lines (THP-1)	XIAP	Drug resistance	(83)
circPAN3		Up-regulated	*in vitro*	AML cell lines (THP-1, K562)	AMPK/mTOR	Induction of drug resistance	(84)
circ-ANXA2	miR-23a-5p, miR-503-3p	Up-regulated	Human, *in vitro*	BM sample, AML cell lines (Kasumi-1, KG-1)		Induction of cell proliferation and drug resistance, inhibition of apoptosis	(85)
circ_PLXNB2		Up-regulated	Human, animal, *in vitro*	BM sample, NOD/SCID mice, AML cell lines (THP1, MV4;11, OCI-AML3, HL-60)	PLXNB2, BCL2, cyclin D1, BAX	Induction of cell proliferation, migration, inhibition of apoptosis	(82)
hsa_circ_0012152	miR-491-5p miR-512-3p miR-4731-5p, miR-296-3p, miR-376c-3p	Up-regulated	Human	BM sample	TP53, MAPK1, PIK3R1, H2AFV, EGFR	Clinical significance	(86)
circ_0012152	miR-625-5p	Up-regulated	Human, *in vitro*	BM sample, AML cell lines (U937 and HL60)	SOX12	Induction of cell proliferation, inhibition of apoptosis	(87)

AML, acute myeloid leukemia; LSC, leukemic stem cell; NOD/SCID, nonobese diabetes/severe combined immunodeficiency; BM, bone marrow.

#### 3.1.1 Tumorigenesis-related CircRNAs

##### 3.1.1.1 Hsa_circ_0000488

Comprehension of a specific sequence, 13q14, which is a region usually removed in leukemia led to identification of hsa_circ_0000488 (circRNA-DLEU2). CircRNA-DLEU2 is a splicing product of the DLEU2 gene (38). DLEU2 gene has been shown to serve as a tumor suppressor gene in hematological malignancies. In adult leukemia, the DLEU2 locus, containing miRNA cluster miR-15a/16-1, is commonly deleted, whereas this region is found to be specifically hypermethylated and transcriptionally repressed in pediatric AML (88). Kasar et al. (89) found that the expression of miR15a/16-1 is negatively regulated through the direct binding of the transcription factor BSAP (B-cell-specific activator protein) to the DLEU2 promoter. Therefore, an increased expression of miR15a/16-1 through DLEU2 promoter depression by histone deacetylation inhibition and BSAP knockdown leads to cell cycle arrest and apoptosis.

While enhanced DLEU2 gene expression may have therapeutic implications, it can also be involved in leukemogenesis. Wu et al. (38) observed markedly up-regulated expression of circRNA-DLEU2 in AML clinical samples and cell lines. Through dual-luciferase reporter and probe assays, miR-496 and its target gene, protein kinase cAMP-dependent catalytic β (PRKACB), were found to be regulated by circRNA-DLEU2, providing mechanistic insight into the role of this circRNA in leukemia biogenesis and progression. MiR-496 antagonized the proliferative and anti-apoptotic effects of PRKACB on leukemia cells. This tumor-suppressive property of miR-496 has already been reported in some studies (90). The PRKACB is a factor which is implies in different cancer development. The PRKACB mRNA and protein levels were decreased in non-small cell lung cancer and exogenous PRKACB impaired the proliferative and invasive ability of LTEP-A2 cells (91). Whereas, in breast cancer cells, PRKACB promoted migration (92). Although these experiments have demonstrated the role of circRNA-DLEU2/miR-496/PRKACB in AML cell activities, such as proliferation, future evaluations are needed to elucidate the *in vivo* role of circ-DLEU2 in the pathophysiology of AML.

##### 3.1.1.2 Hsa_circ_100290

According to the circRNA microarray expression dataset GSE94591, hsa_circ_100290 expression is highly up-regulated in AML samples (55). A previous study identified hsa_circ_100290 regulatory functions in human oral squamous cell carcinomas (OSCC). Hsa_circ_100290 combines explicitly with the miR-29b family as a ceRNA to regulate the expression of CDK6 by acting as a miRNA sponge (93). Fan et al. (55) reported that hsa_circ_100290 could promote leukemia cell proliferation but inhibited apoptosis by regulating the expression of cyclin D1, CDK4, Bcl-2, and cleaved caspase-3. CyclinD1/CDK4 axis is critical in driving cell-cycle progression, which promotes G1/S transition, and Bcl-2 and cleaved caspase-3 are significant effectors of mitochondrial apoptosis (94, 95). Mechanistic analysis showed that hsa_circ_100290 might act as a ceRNA of miR-203 to relieve the repressive effect of miR-203 on its target Rab10. In previous studies, miR-203 was reported to exhibit anti-tumor effects on various cancers, including hepatocellular carcinoma, esophageal, colorectal, and prostate cancers (96–102). In leukemia, K562 leukemia cells transfected with a hsa-miR-203 plasmid were sensitized to arsenic trioxide by inducing apoptosis and reducing BCR/ABL gene expression levels (103).

Rab10 is a member of the RAS superfamily of small GTPase, which exhibit oncogenic activity in esophageal squamous cell carcinoma, osteosarcoma, and hepatocellular carcinoma (104–107). Luciferase reporter assays showed that Rab10 was the direct target of miR-203. Hsa_circ_100290 knockdown suppressed leukemia cell proliferation by induction of G1 phase arrest and enhanced apoptosis by decreasing the expression of Rab10, while the effects of hsa_circ_100290 knockdown on the level of protein expression is rescued by the transfection of miR-203 inhibitors (55).

##### 3.1.1.3 Hsa_circ_0000370

The main subgroups of AML involve a large variety of genetic alterations, including amplification, deletions, rearrangements, and point mutations (108). Mutations of the FMS-like tyrosine kinase 3 (FLT3) and internal tandem duplication (ITD) are common driver mutations in AML. Recently, some studies have proved the miRNA involvement in various aspects of the pathogenesis of FLT3-ITD+ AMLs, including cell proliferation, apoptosis, and resistance to chemotherapy (109, 110). For example, in FLT3-ITD AMLs, the miR-155/Nf- κB/STAT5 regulatory network targets myeloid transcription factor PU.1. accelerates proliferation and inhibits apoptosis; miR-451a sensitized FLT3-ITD-associated leukemic cells to chemotherapy by targeting multidrug resistance protein 1 (MDR1) (111).

Zhang et al. (58) screened the circRNA expression profile in AML samples using microarray analysis. They found that circ_0000370 expression was markedly up-regulated in FLT3-ITD+ AML samples. Circ_0000370 is originated from the FLI-1 gene and regulated by FLT3-ITD. A significant increase in circ_0000370 was observed when FLT3-ITD was overexpressed, and both quizartinib and gilteritinib (FLT3 inhibitors) decreased circ_0000370 expression level. The researchers found that overexpression of circ_0000370 increased cell viability and inhibited apoptosis, whereas circ_0000370 knockdown presented the opposite of these results. Mechanistically, circ_0000370 acted as a sponge of miR-1299 and then decreased its expression. MiR-1299 was previously reported to exert tumor-suppressive activity. MiR-1299 negatively regulates STAT3, thus inhibiting the colon cancer cell growth and inhibiting HCC cell proliferation by targeting CDK6 (112, 113). Besides miR-1299, circ_0000370 targets miR-370-3p, miR- 502-5p, miR-640, and miR-1281 which are closely related to signaling pathways in cancer. S100A7A is a gene target of circ_0000370 identified by Bioinformatics analysis. S100A7A is a member of the S100 protein family, which acts as a chemotactic agent and induces the secretion of proinflammatory cytokines in AML (114).

##### 3.1.1.4 Circ-MYBL2

The other circRNA which its functional significance was assessed in the context of the AML with FLT3-ITD mutations was circ-MYBL2. Despite the utility of the number of tyrosine kinase inhibitors (TKIs) that block the oncogenic signaling triggered by FLT3, primary and secondary acquired resistance to FLT3 inhibitors, such as quizartinib, remains a challenge in FLT3-ITD AML treatment (115). Nonetheless, circRNAs have been ideal therapeutic targets to tackle this problem. Circ-MYBL2 is a circular form of MYBL2, a cell cycle checkpoint gene. Sun et al. (56) discovered that circ-MYBL2 is significantly up-regulated in FLT3-ITD^+^ AML compared to wild-type FLT3 AML. Circ-MYBL2 promotes proliferation and cell-cycle progression and inhibits apoptosis of FLT3-ITD^+^ leukemia cells. Circ-MYBL2 knockdown in leukemia cells harboring the FLT3-ITD mutation increased the number of cells in Sub-G1 and G0/G1 phases and decreased ones in S and G2/M phases, thus substantially impairing the proliferation of these cells, whereas it merely affected the FLT3-nonmutant cells. Conversely, on the knockdown of circMYBL2, the apoptotic activity of FLT3-ITD^+^ AML cells increased, while no effect was observed in FLT3-ITD^-^ AML cells. Notably, similar cytoactivity impairment was observed upon circ-MYBL2 knockdown in quizartinib-resistance cells.

Further, it was shown that circ-MYBL2 recruits and directly interacts with polypyrimidine tract-binding protein 1 (PTBP1), resulting in strongly enhanced translation of FLT3 kinase (56). PTBP1, a posttranscriptional gene expression regulator, modulates mRNA metabolism, particularly its translational regulation (116). A loss of circ-MYBL2 in mutant cells resulted in a decline in FLT3 phosphorylated kinases and impairment of STAT5 phosphorylation, a key downstream protein of oncogenic FLT3 and crucial for aberrant leukemia cell growth. Likewise, circ-MYBL2 knockdown decreased the expression of the STAT-5-dependent protein c-MYC in both cell lines and patients’ samples. Previous studies have shown that FLT3-ITD mutation silence p27/Kip1 and promotes MCL-1 expression to accelerate cell cycle progression and induce apoptosis resistance (117, 118), while circ-MYBL2 knockdown counteracts these effects. *In vivo* findings further showed that circ-MYBL2 promotes the leukemogenesis and infiltration of FLT3-ITD^+^ AML cells. Also, the overall survival time of circ-MYBL2 knockdown animals was longer than those with high expression of circ-MYBL2.

##### 3.1.1.5 Circ-NPM1

Alternative and aberrant splicing events are common characteristics of AML involving many genes in patients with AML. They might also impact patients carrying a nucleophosmin 1 (NPM1) gene mutation. NPM1 mutations constitute the most common genetic lesion in adult AML. Given that an atypical splicing event are generate the circRNA-forming exons and general deregulation of splicing mechanisms in AML, the circRNA expression profile is also impaired, and such altered circRNAs can be functionally relevant and contributes to leukemogenesis. Thus far, 29 NPM1 circRNA variants are identified according to information provided in the AceView and circBase databases (119). In addition to known circular NPM1 transcripts, Hirsch et al. (39) provided evidence for a novel variant, hsa_circ_0075001, with a highly differential expression pattern in leukemia cell lines. Moreover, when examining circRNA expression in AMLs of various subgroups, a significant difference was reported, with no or minimally differentiated subgroups [M0 or M1 subtypes based on the French-American-British (FAB) classification system] exhibiting higher levels of hsa_circ_0075001 as compared to their counterpart mature forms (M2, M4, and M5). Furthermore, a positive correlation exists between hsa_circ_0075001 expression and total NPM1 expression, regardless of NPM1 mutation status.

A comparative gene expression pathway analysis showed that an up-regulated level of hsa_circ_0075001 is strongly connected with the down-regulation of genes involved in the TLR pathway, specifically TLR1 and TLR4, TLR5, TLR7, and TLR8, together with CD14 and MYD88 (39). In addition to TLRs’ notable role in the innate immunity and inflammatory responses, they were critical in the development of hematopoietic and other malignancies. Indeed, aberrant TLR expression has been associated with LSCs survival (120). Interestingly, 185 genes differentially expressed by patients with high hsa_circ_0075001 are miR-181 targets. The latter plays a role in cytogenetically-normal leukemia (CN-AML) (121). Interestingly, Marcucci et al. (122) showed an inverse correlation between expression levels of miR-181 family and genes involved in pathways of innate immunity mediated by TLRs and interleukin (IL)- 1β. Since the NPM1 gene contains binding sites for miR-181, it warrants further investigation of the possible interaction between circular NPM1 and the miR-181 family. Ding et al. (40) reported that circ-NPM1 was an AML chemoresistance mediator. Functional studies have shown that inhibition of malignant behavior of AML cells and ADM resistance by circ-NPM1 was achieved *via* the miR-345-5p/FZD5 axis. NPM1 was an endogenous sponge for miR-345-5p to up-regulate frizzled-5 (FZD5).

Downregulation of circ-NPM1 or up-regulation of its binding miR-345-5p suppressed cell cycle progression, colony formation, and the capacity of migration and invasion and increased apoptosis rate and sensitivity of leukemia cells to leukemia cells Adriamycin (ADM). MiR-345-5p was previously reported to act as a tumor-suppressor, restricting AML cell proliferation and facilitating apoptosis through regulating PI3K/AKT signaling pathway (123). Potential tumor-promoting roles of FZD5 were previously reported in various cancers, such as proliferation, invasion, angiogenesis, and chemoresistance after cancer recurrence (124). These findings indicate circ-NPM1 degradation could have a therapeutic effect in AML resistance therapy which can brought a promising future in the field.

##### 3.1.1.6 FP-circRNAs: f- circPR, f-circM9, and circAF4

Chromosome translocations are often early or initiating events in leukemogenesis characterized by juxtapositioning and illegitimate fusion of two separate regions of the genome. In addition, common products of chromosome translocations and fusion genes, recent studies indicate that chromosome translocations associated with cancers may increase fusion- circRNA (f- circRNA). Few f-circRNAs have been described in hematological malignancies such as AML, chronic myelocytic leukemia (CML), and anaplastic large cell lymphoma (ALCL). Guarnerio et al. (59) found that two well-established leukemia-associated chromosomal translocations, t (15,17) (q24;q21) and t (9,11)(p21;q23), lead to the expression of f- circRNAs specific to the breakpoint junction. The t (15,17) (q24;q21), generating the chimeric fusion gene PML-retinoic acid receptor a (PML-RARa), is the hallmark of acute promyelocytic leukemia (APL). Molecular analysis in patients with PML-RARa translocation and APL-derived leukemic cell line revealed that the PML-RARa fusion gene could produce one or more f- circRNAs named f-circPR. The t (9,11) (p21;q23) fuses the MLL gene with MLLT3 (also known as AF9 or LTG9) and is the most common MLL translocation producing AML. This chimeric fusion gene produces two f- circRNAs products, f- circM9-1 and f- circM9-2. In mouse embryonic fibroblasts, f-circPR and f-circM9_1 have been shown to increase cell proliferation, inhibit apoptosis, and confer therapeutic resistance to arsenic trioxide (ATO), thus promoting leukemia progression regardless of their linear transcript and their fusion protein counterparts. About 10% of all leukemias harbor MLL1 translocations (125). Until today, totally 135 different rearrangement of MLL have been determined; the MLL gene can be fused with one of more than 100 partners, including AF4, AF6, AF9, AF10, EPS15, and ENL, to generate over 100 MLL fusion genes (126). In a large circRNA profiling study, Huang et al. (60) examined these 6 MLL rearrangements; MLL-AF4, MLL-AF10, MLL-ENL, MLL, AF9, MLL-AF6, MLL-AF10, which are the most common among them, and MLL-GAS7, which constitute about 60% of all acute leukemia harboring MLL rearrangement. These fusion gene partners generated several circRNAs, referred to fusion gene partners-originating circRNAs (FP-circRNAs). Further, these researchers identified and characterized a circRNA derived from the MLL fusion partner AF4 gene, called circAF4, which functions as an oncogene and promotes leukemogenesis. CircAF4 expression positively correlates with MLL-AF4 protein levels. Regarding the mechanism, previous studies have shown that the direct target of tumor-suppressor miR-128-3p is the AF4 gene. MiR-128-3p and its validated target genes are involved in several tumorigenic molecular pathways in many cancers, such as prostate cancer, lung cancer, glioma, colorectal cancer, breast cancer, and MLL-AF4 acute lymphocytic leukemia (ALL). CircAF4 sponges miR-128-3p to up-regulate the MLL-AF4 expression. CircAF4 knockdown could increase the apoptotic activity of leukemia cells and delay the progression of aggressive leukemia *in vitro* and *in vivo* (60). Taken together, these studies illustrate the oncogenic roles of f-circRNAs in leukemias harboring specific genetic alterations and the therapeutic potential of disruption of their expression.

##### 3.1.1.7 CircSPI1

CircSPI1 originated from the lineage-determining gene SPI1 (61). The SPI1 gene encodes a transcriptional factor called PU.1, one of the most critical regulators of normal hematopoiesis, particularly myeloid differentiation (127). Dysregulation of PU.1 has been described in most leukemia types (128). Wang et al. (61) reported circSPI1, with its main expression to be in hematopoietic malignancies, presents a hematopoietic lineage-specific expression. CircSPI1 is increased in AML and plays a part in leukemogenesis by antagonizing SPL1 and interacting with several miRNAs. Specifically, APL and AML with normal karyotype exhibit higher levels of circSPI1. Next, the researchers showed that knockdown of circSPI1 inhibited cell proliferation, induced partial myeloid differentiation, and enhanced apoptosis of leukemia cells through upregulation of the levels of expression of apoptosis-related proteins such as Bcl-2, CDK6, p-ERK1/2. Notably, circSPI1 exerts these effects antithetically and independently to linear SPI1, a tumor suppressor. One thousand three hundred ninety genes were differentially expressed upon circSPI1 knockdown. Functional enrichment analysis further revealed that a fair number of these circSPI1-related genes, such as IRF8, ICAM1, HOXA9, and CDK6, have mainly been involved in leukemic transformation and leukemia cells maintenance. CircSPI1 also antagonized SPI1 through binding to the transcription initiation factor elF4AIII to modulate myeloid differentiation. Furthermore, bioinformatics and luciferase assays confirmed that miR-1307-3p, miR-382-5p, and miR-767-5p were targeted by circSPI1. Furthermore, the target genes of these miRNAs are implicated in several critical cancer signaling pathways, including Ras, PI3K/Akt, p53, JAK/STAT, and FOX5 (61). These findings highlight the significance of circRNAs that originate from the genetic loci encoding hematopoietic transcription factors.

##### 3.1.1.8 Hsa_circ_0001346

Zhang et al. showed that hsa-circ-0001346 (circRNF13) was a circRNA with tumorigenic potential, which is up-regulated in AML patients (62). CircRNF13 is derived from the RING finger protein (RNF13) amplicon. Aberrant expression of circRNF13 has been reported in lung adenocarcinoma, and circRNF13/miR-93-5p/Apo2 regulatory network is involved in tumor invasion and metastasis (129). CircRNF13 promotes the malignant evolution of AML by acting as a sponge for miR-1224-5p. Downregulation of miR-1224-5p has been reported in many types of cancers. For instance, miR-1224-5p suppressed migration, invasion, and epithelial-mesenchymal transition (EMT) in colorectal cancer by targeting the SP1-mediated NF-κB signaling pathway (130). The proliferative assay showed that circRNF13 knockdown repressed the AML cells proliferation and down-regulated C-myc expression, an essential regulator of cellular proliferation.

Further, the number of colonies formed in circRNF13 low expression cell lines was significantly reduced. Apoptosis assay indicated that circRNF13 knockdown blocked the cell cycle at G0/G1 and increased the apoptosis rate by activating Caspase 3/7. Notably, both migration and invasion were attenuated by circRNF13 downregulation. CircRNF13 mediates these by inhibiting the Tenascin-C expression, an extracellular matrix glycoprotein affecting cell migration and tumor invasion (62).

##### 3.1.1.9 Hsa_circ_0004136

As pediatric leukemia carries its unique biological features, some studies also functionally characterized the circRNA expression in childhood leukemia. Circ_0004136 and circ_0005774 are two of the top 20 differentially expressed circRNA in pediatric AML patients, and their function and mechanism in leukemogenesis were investigated. Circ_0004136 could significantly enhance the progression of pediatric AML by binding to miR-142 and miR-570-3p, thus modulating the expression of their respective target genes. Yuan et al. screened the circRNAs expression pattern in pediatric AML by circRNA microarray analysis and revealed 569 circRNAs were differentially expressed with 273 circRNAs up-regulated and 296 circRNAs downregulated. Among them, circ_0004136 was the most up-regulated circRNA. AML cell proliferation is significantly reduced upon inhibition of circ_0004136. Using miRanda and TargetScan algorithms, several miRNAs, including miR-29a, miR-196a, and miR-142-3p, were regulated by circ_0004136 (63). The clinical significance of dysregulation of these miRNAs has been previously reported in pediatric and adult leukemia (131). For instance, a Low level of miR-29a was associated with aggressive tumor progression and unfavorable prognosis in pediatric AML (132). In contrast, upregulation of miR-196a was associated with poor prognosis in adult AML (133). MiR-142 is highly expressed in the hematopoietic system, regulates the differentiation and function of hematopoietic cells, and contributes to leukemic transformation (134). Circ_0004136 promotes cellular proliferation by sponging miR-142.

In another study, Jing et al. reported that the upregulation of circ_0004136 in extracellular vesicle (EVs)-derived from pediatric AML patients contributes to the significant progression of the disease. Circ_0004136 functions as a miR-570-3p sponge and promotes leukemia through positive regulation of miR-570-3p target, tetraspanin3 (TSPAN3) (64). Previous studies have shown the critical role of TSPAN3 in the development and propagation of AML (135). EVs containing circ-0004136 can maintain cell viability and promote autonomous proliferation in AML cells. In contrast, exosome-mediated circ_0004136 knockdown inhibited AML-related pathological processes, including viability, cell cycle progression, migration, and invasion but enhanced cell apoptosis.

##### 3.1.1.10 Circ_0005774

Circ_0005774 is another deregulated circRNA in pediatric leukemia. Li et al. (65) showed that knockdown of circ_0005774, on the one hand, could inhibit cell proliferation and, on the other hand, could promote apoptosis as indicated by lower cell viability, decreased proliferation markers like proliferating cell nuclear antigen (PCNA), CyclinD1, and Bcl-2 expression, as well as increased apoptosis rates. Circ_0005774 exerted these functions by targeting miR-192-5p, resulting in uncoordinated 51-like kinase 1 (ULK1) expression. Recent studies have shown a close relation between miR-192-5p and various pathological and physiological processes, particularly with cancer-related biological pathways (136, 137). According to Gene Expression Omnibus (GEO) database, blood samples from AML patients showed aberrant expression of miRNAs. MiR-192-5p was one of these miRNAs, in further evaluation the highest level of decreased expression by qRT-PCR assay was showed (138). However, miR-192-5p expression was higher in patients with relapsed APL than in newly diagnosed patients (139). A positive correlation was observed between miR-192-5p expression level and OS and EFS in patients (140). As expected, miR-192-5p overexpression suppressed tumor cell proliferation while enhancing cell differentiation and apoptosis (141). ULK1 is considered a key protein in the regulation of autophagy initiation. ULK1 aberrant expression is associated with the progression of various solid tumors and poor prognosis (142, 143). In this study, the authors also claimed that ULK1 is a novel downstream target of miR-192-5p. Recently, it was reported that inhibition of ULK1 induced leukemia cell death and could be a therapeutic approach in AML treatment (144, 145). This finding may lead to improved understanding of ceRNA networks and their leukemogenic properties.

##### 3.1.1.11 Hsa_circ_0002483

Hsa_circ_0002483 is a tumor-promoting circRNA that is up-regulated in AML patients (68). However, circ-0002483 was previously demonstrated to suppress progression and enhance chemosensitivity of non-small-cell lung carcinoma (NSCLC) *via* sponging miR-182-5p (146). Different tumor microenvironments could explain these dual functions of circ-0002483. In a study by Xiao et al. (68) silencing circ_0002483 by siRNA delivery was shown to suppress AML cell proliferation and cell cycle progression at G0/G1 phase. Further, balance between pro-apoptotic Bax and anti-apoptotic Bcl-2 proteins is impaired by circ_0002483 downregulation; this reduction in Bax/Bcl-2 ratio also decreases the activity of C-caspase-3 eventually leads to cell apoptosis. Through dual-luciferase reporter assay, miR-758-3p was verified as the target of circ_0002483. Thus, up-regulated circ_0002483 functioned as an oncogene in AML through the downregulation of miR-758-3 which represses leukemia cell growth by targeting MYC. In AML, MYC oncogene aberrantly activates and has a crucial role in in inducing leukemogenesis. Additionally, this leukemogenic activity might be modulated by a non-coding RNA network (116, 147). MiR-758-3 is downregulated in many cancer types, and it was therefore implicated as a tumor suppressor. In a recent study, lncRNA SNHG3 facilitated AML cell growth by sponging miR-758-3 to up-regulate SRGN, suggesting the anti-leukemic function of miR-758-3 (148). These findings suggest that hsa_circ_0002483/miR-758-3/MYC axis is critical for AML progression, providing a new potentially therapeutic approach for AML patients.

##### 3.1.1.12 Hsa_circ_101141 (circ-ANAPC7)

By screening the circRNA profiles of 85 patients diagnosed with AML, Chen et al. characterized 698 differentially expressed circRNAs. Circ-ANAPC7 was one of these circRNAs that showed the highest increased expression level in further evaluation by qRT-PCR assay. According to bioinformatics analysis, circ-ANAPC7 contained miRNA response elements (MREs) for several miRNAs, including the miR-181 family, hsa-miR-338-3p, and hsa-miR-526b-5p. Among these miRNAs, the miR-181 family play a prominent role in AML development. In GO and KEGG pathway enrichment analysis, the circ-ANAPC7/miR181 axis is closely linked with several signaling pathways regulating AML cell activity and particularly leukemogenesis (67). The miR-181 family is among the miRNAs first identified as hematopoietic lineage-specific miRNAs and are implicated in regulating cellular differentiation, specifically cells of hematopoietic origin. Meanwhile, aberrant expression of these miRNAs has been reported in many types of tumors, including leukemia. There is some evidence that miRNA-181 dysregulation influences AML pathogenesis and prognosis (149). In a study by Huang et al., miR-181a attenuates AML aggressiveness through the RAS/MAPK pathway and represents a potential clinical application (150). Furthermore, a recent study by Shen et al. showed that circ-ANAPC7 could serve as a promising biomarker in the monitoring of AML. The area under the curve (AUC) for assessing the prognostic power of circ-ANAPC7 revealed a 0.915 value suggesting its significant value in auxiliary AML diagnosis. Circ-ANAPC7 expression is markedly richer in newly diagnosed and relapsed AML patients, and its expression was correlated with peripheral white blood cell (WBC) counts and BM blast percentage (151).

##### 3.1.1.13 Circ-0009910

Circ-0009910 is derived from the MFN2 gene and is one of the well-studied circRNAs acting as a sponge for several miRNAs. Circ-0009910 has been found to be involved in the progression and tumorigenesis of various cancers. For example, upregulation of circ-0009910 in ovarian cancer indicates poor prognosis and promotes cancer cell phenotypes by targeting miR-145 (152). Also, overexpression of circ-0009910 promotes osteosarcoma progression by activating the JAK1/STAT3 signaling pathway through regulation of the miR-449a/IL6R axis (153). Regarding hematological malignancies, circ-0009910 was found to be overexpressed in serum of imatinib-resistance patients and cell lines and accelerates imatinib-resistance in CML cells by sequestrating miR-34a-5p (154). Circ-0009910 was up-regulated in AML patients compared with iron- deficiency anemia patients. Also, the OS rate was lower in patients who had high expression of circ-0009910 in comparison to those with low expression. Circ-0009910 is able to induce apoptosis in AML cells by sponging miR-20a-5p and knocking it down (70). MiR-20a-5p is a double face gene and overexpression of this miRNA was putatively involved in the cancer-related pathological process such as proliferation, invasion, and metastasis and radio-resistance in breast cancer, HCC, nasopharyngeal and colorectal cancer (155–158). In contrast, it is downregulated in osteosarcoma and neuroblastoma (159).

Circ-0009910 expression was up-regulated in AML clinical specimens and cell lines, even higher in AML-derived exosomes. Exo-circ-0009910 expression level was correlated with the patient’s risk stratification. Furthermore, molecular mechanism research showed that exo-circ-0009910 and intercellular circ-0009910 promote leukemia cell proliferation and cell cycle transition by up-regulating growth factor receptor-bound protein 10 (GRB10) *via* sponging miR-5193-3p. Consistent with its role in promoting cell proliferation, circ-0009910 knockdown increased apoptosis through modulating the Bcl-2/Bax pathway (71). Tumor suppressive roles of miR-5193-3p were also demonstrated in other malignancies, and it was through regulating the TGF-β signaling pathway and targeting key oncogenes (160, 161). In keeping with these reports, Wu et al. (72) found that the level of circ-0009910 was increased in AML clinical specimens and cell lines. Mechanistically, circ-0009910 targeted miR491-5p, and its regulatory function was related to miR491-5p sponging. MiR491-5p inhibitor abrogates the inhibitory function of circ-0009910 knockdown. MiR491-5p has been deregulated in several human cancers (162). Similarly, contributing role of LncRNA VPS9D1/miR491-5p-miR-214-3p/GPX1 axis in ALL progression was previously reported (163). Moreover, circ-0009910 was found to regulate β-1, 4-Galactosyltransferase gene (B4GALT5) level and activate PI3K/AKT signaling pathway by targeting miR491-5p. B4GALT family has been recognized to mediate chemotherapy resistance in leukemia cells (164).

##### 3.1.1.14 Hsa_circ_0079480

Another example of circRNA-miRNA-mRNA regulatory network in AML, is circ_0079480/miR-654-3p/HDGF axis. Hu et al. (69) identified the role of hsa_circ_0079480 that was up-regulated in AML cells. These researchers found that the circ_0079480 knockdown decreased leukemia cells’ viability and proliferative ability. Also, research showed evidence of circ_0079480 acting as a miRNA sponge and decreasing the expression level of miR-654-3p. MiR-654-3p is been reported to be a tumor suppressor gene in HCC, gastric cancer, and prostate cancer, influencing cancer cells’ progression, invasion, and migration (165–167). In addition, circHIPK3 is involved in glioma development by binding to miR-654-3p in a ceRNA manner (168). MiR-654-3p inhibits the expression of the hepatoma-derived growth factor (HDGF), which has oncogenic properties in several malignancies. For instance, downregulation of HDGF inhibits bladder cancer cell development by inactivating the PI3K/AKT signaling axis (169). In another study, lncRNA AGAP2AS1 promoted glioma cell progression by regulating the miR-15a/b-5p/HDGF/Wnt/β-catenin axis (170). Nonetheless, the activity of HDGF in AML needs further clarification. This study saw a reverse correlation between HDGF expression level and OS in patients.

##### 3.1.1.15 Circ_TASP1

Circ_TASP1 was also elevated in AML tissues and cells and its high expression correlated with a poor prognosis. Moreover, the knockdown of circ_TASP1 significantly suppressed the proliferation and apoptosis of AML cells. Mechanistically, suppression of circ_TASP1 enhanced miR-515-5p expression and decreased the expression of its downstream target, high mobility group A2 (HMGA2) oncogene. HMGA2 played an essential role in driving AML progression potentially through activating the Wnt/β-catenin signaling. Notably, depletion of circ_TASP1 suppressed tumor growth *in vivo* (78).

##### 3.1.1.16 Circ_PTK2

Hsa_circ_104700/hsa_circ_0005273 (circ_PTK2) is another example of up-regulated circRNAs in AML, enhancing the expression of forkhead box M1 (FOXM1) through sequestrating miR-33-5p. The proliferation of leukemia cells and inducing apoptosis has been inhibited by Circ_PTK2 silencing with reducing cyclin D1 expression and Bcl-2/Bax level regulation. Higher expression of circ_PTK2 can predict reduced survival time and probably be used as a marker to determine AML prognosis (79). Circ_PTK2 had been previously identified as an oncogene in thyroid carcinoma through regulating SOX2 (171).

##### 3.1.1.17 Other circRNAs

Hsa_circ_0003602 was up-regulated in AML clinical specimens and cell lines, and its overexpression correlated with poor survival. Hsa_circ_0003602 can serve as competitive endogenous RNA (ceRNA) to up-regulate insulin-like growth factor 1 (IGFR1) by sponging miR-502-5p and enhancing AML cell proliferation, migration, and invasion. IGFR1 gene deregulation has been known to mediate several aspects of the malignant phenotype and plays a critical role in regulating the LSCs proliferation (77). Similarly, circ_0058058, circ_SFMBT2, and circ_ATAD1 are up-regulated in AML and have been demonstrated to promote cell growth and proliferation *via* the miR-4319/eukaryotic imitation factor 5A2 (EIF5A2), miR-582-3p/zinc finger and BTB domain containing 20 (ZBTB20), miR-34b regulatory axes, respectively (80–82). In turn, silencing their expressions or up-regulating the expression of their target miRNAs effectively suppresses the malignant behavior of AML cells and accelerates apoptosis.

#### 3.1.2 Tumor-Suppression-Related circRNAs

##### 3.1.2.1 Hsa_circ_0121582

Down-regulated circRNAs pose anti-oncogenic characteristics, inhibiting proliferation, cell cycle progression, migration, and invasion of AML cells. They act as ceRNAs similarly to those that promote tumor growth. Inhibited miRNAs act primarily as oncogenes that stimulate tumor-stimulating genes or inactivate related tumor suppressors. The miRNA mediator primarily regulates the expression of a downstream protein and alters a signaling pathway through a signaling axis. Chen et al. (43) conducted high-throughput sequencing technology and confirmatory qRT-PCR to screen dysregulated circRNAs in AML. Circ_0121582 has been shown to be significantly downregulated in AML clinical specimens and cell lines. Circ_0121582 is a splicing product of the GSK3β gene and is positively correlated with the cognate linear GSK3β RNA expression.

Circ_0121582 has been shown to arrest leukemic cell proliferation and DNA synthesis and raise the number of leukemic cells in the G0/G1 phase. *In vivo* experiments showed that the weight and volume of xenograft tumors were lower in circ_0121582-overexpressing animals than in those in the control group. Additionally, circ_0121582 exerted a repressive effect on leukemia growth by promotion of GSK3β expression through sponging miR-224 and direct binding to ten-eleven translocation 1 (TET1). GSK3β is inactivated due to promoter methylation and act as a tumor suppressor gene.

Circ_0121582 introduces DNA methylase TET1 to the GSK3β promoter, thus enhancing its transcription (43). GSK3β is a negative regulator of the Wnt/β -catenin pathway. This signaling pathway is involved in the pathogenesis of AML through several mechanisms and is crucial for the maintenance of leukemia stem cells (LSCs) (172).

##### 3.1.2.2 Circ_0001947

Using microarrays, Han et al. (173) showed a differential expression of several circRNAs in newly diagnosed AML patients before treatment compared to healthy controls. They selected two candidate circRNAs out of 464 dysregulated circRNAs, up-regulated hsa-circ-0058058 and downregulated hsa-circ-0001947. Subsequently, they validated the microarray results of these two circRNAs using qRT-PCR and observed that circ_0001947 expression was downregulated in BM samples of newly diagnosed and relapsed-refractory patients, while patients achieved CR exhibited a higher level of circ_0001947 expression. Circ_0001947 was generated from the AFF2 gene and found to be positively correlated with the expression of the AFF2 mRNA. In addition, the AUC values of circ_0001947 in the ND stage, CR stage, and relapsed-refractory (RE) stage were 0.8911, 0.9057, and 0.7989, respectively. By applying different prediction tools, three hsa-miR-329-5p, hsa-miR-10b-3p, and hsa-miR-488-3p exhibited the most potent circRNA-miRNA-mRNA gene interaction networks. CREBRF, KLHL15, KRT12, and INTS9 were among the hundreds of mRNA targets of these top three miRNAs targeted by more than one of them.

GO, and KEGG pathway enrichment analyses showed that these target genes are involved in several biological processes, including multicellular organism growth, intracellular signal transduction, and activation of protein kinase B. Mechanistically, circ_0001947 serves as a molecular sponge of miR-329-5p and inhibits the miR-329-5p mediated targeting repression of the CREBRF AML suppressor gene. Studies have identified crucial roles of miR-329-5p in immune-related and apoptosis pathways. Additionally, several studies confirmed that CREBRF regulates angiogenesis by high-density lipoproteins (174). CREBRF is also known to promote the proliferation of human gastric cancer cells *via* the AKT signaling pathway (175). Previous studies have also shown that CREBRF was a tumor suppressor in glioblastoma, inhibiting hypoxia-induced autophagy through the CREB3/ATG5 pathway (176). Notably, *in vivo* downregulation of circ_0001947 stimulated cell proliferation and alleviated cellular apoptosis, thereby promoting AML progression. Regulation of Bcl-2, c-Myc, and caspase1/9 mediated these effects.

##### 3.1.2.3 Circ_0040823

Circ_0040823 was downregulated in AML samples and cell lines. Also, patients with high expression of circ_0040823 had poorer OS and DFS rates than those with low expression. Consistent with expectation, circ_0040823 overexpression efficiently inhibited leukemia cells proliferation and cell cycle progression while silencing circ_0040823 expression enhanced cellular proliferation. The upregulation of circ_0040823 also repressed leukemia tumor growth *in vivo*. Moreover, miR-516b can be directly combined with circ_0040823 and PTEN mRNA. Upregulation of circ_0040823 increased the expression of PTEN, while downregulation of PTEN was observed with miR-516b overexpression. Cellular apoptosis was induced when miR-516b inhibition downscaled leukemia cells proliferation and cell cycle progression (74). Previous studies have demonstrated that the expression level of miR-516b is decreased in a number of tumors, such as lung cancer and esophageal squamous cell carcinoma (177, 178). In contrast, in AML, miR-516b expression is up-regulated and plays an oncogenic role (74). PTEN is a tumor suppressor that regulates various cancer-related biological processes such as cellular proliferation, migration, self-renewal, and metabolism (179). PTEN expression was significantly lower in BM of AML patients (180). Additionally, deletion, mutations, transcriptional silencing, and protein inactivation cause PTEN to be functionally lost in human leukemias (181). These data indicated that the circ_0040823/miR-516b/PTEN axis might be a therapeutic target against AML.

##### 3.1.2.4 Circ_CRKL

Circ_CRKL is produced from V-Crk Avian Sarcoma Virus CT10 Oncogene Homolog-like (CRKL). Although, circ_CRKL possesses tumor suppressor roles in AML, its parent gene, CRKL, has been characterized as an oncogene. Liu et al. (75) observed that circ_CRKL overexpression remarkably suppressed AML cell proliferation, arrested cell-cycle progression, and reduced the colony-forming capacity of AML cells. Mechanistically, circ_CRKL exerts these anti-leukemic functions by sponging miR-196a-5p and miR-196b-5p to regulate P27. P27 is a cyclin-cyclin-dependent kinase (CDK) inhibitor that mediates cell-cycle arrest (182). Cell cycle-related proteins, such as p27, have attracted attention due to the indefinite proliferation of cancer cells. Consequently, cancers with abnormal p27 metabolism/localization are characterized by poor prognosis and response to treatment. P27 functions and cellular localization are mainly determined by post-translational modifications, such as amino acid phosphorylation (183). However, this study provides evidence that P27 could also regulate at the posttranscriptional level by circ_CRKL.

##### 3.1.2.5 Circ_0003420

LSCs are a small subset of leukemia cells that can initiate leukemogenesis and promote leukemia progression. Despite the potential roles for circRNAs in AML, only a few studies have investigated the possible relation between circRNAs and the acquisition of SC-like characteristics in AML. Circ_0003420 is up-regulated in liver tissue samples from patients with sepsis and *in vitro* model of sepsis-induced liver damage, and knocking it down alleviates sepsis-induced liver damage *via* targeting NPAS4 (184). In a study by Lin et al. (76), circ_0003420 expression was significantly downregulated in LSCs (CD34^+^CD38^-^ cell population) of AML cell lines and poor prognosis patients. Also, circ_0003420 expression was higher in patients who achieved CR following cytarabine and daunorubicin therapy, indicating this circRNA could serve as a prognostic marker. *In vivo* studies showed that circ_0003420 overexpression reduces the survival rate of leukemia cells, which further led to leukemogenesis inhibition. Overexpression of circ_0003420 considerably inhibited the proliferative ability and viability of leukemia cells but promoted cell apoptosis through regulation of Bcl-2/Bax ratio and cleaved caspase-3. It has been shown that circ_0003420 overexpression influenced the levels of three primary markers of LSC phenotype, ABCB1 transporter, CD34, and MMRN1. Further molecular mechanism research revealed that circ_0003420 sponged the mRNA of IGF2BP1 and markedly repressed its levels within LSCs (76). The IGF2BP1 gene is highly expressed in various human tumors, including lung, melanoma, breast, colon, and hepatocellular tumors, and is linked to poor outcomes and cancer metastasis. In leukemia, IGF2BP1 regulates several key stem-cell regulatory factors, including HOXB4, MYB, and ALDH1A1, which affect the proliferation and tumorigenic ability of LSCs (185). These data exemplified the multi-mechanistic roles of circRNAs in AML progression.

### 3.2 Clinical significance of circRNAs in diagnosis, prognosis and therapy of AML

As previously mentioned, leukemias are a group of disorders characterized by the accumulation of undifferentiated myeloid cells. The leukemic clone has a competitive advantage and can impair normal hematopoiesis leading to marrow failure and causing sustained cytopenia and immunodeficiency. Treatment options for AML vary based on the leukemia type and the person’s comorbid conditions, including general supportive therapy, cytotoxic regimens, targeted therapies, and allogeneic hematopoietic stem cells transplantation (allo-HSCT) (10). AML patients are generally assigned to these therapies based on prognostic factors such as age, genetic abnormalities, and comorbidities. Majority of patients with AML are assigned to intermediate and adverse risk groups with unfavorable cytogenetics (mutations and gene fusions) that cannot be treated with conventional chemotherapy agents. Typically, these patients undergo allogeneic SCT when eligible (donor availability and comorbidities) (186). Therefore, an urgent need exists to determine powerful biomarkers in determining the prognosis and early diagnosis of AML.

**Figure 3:**
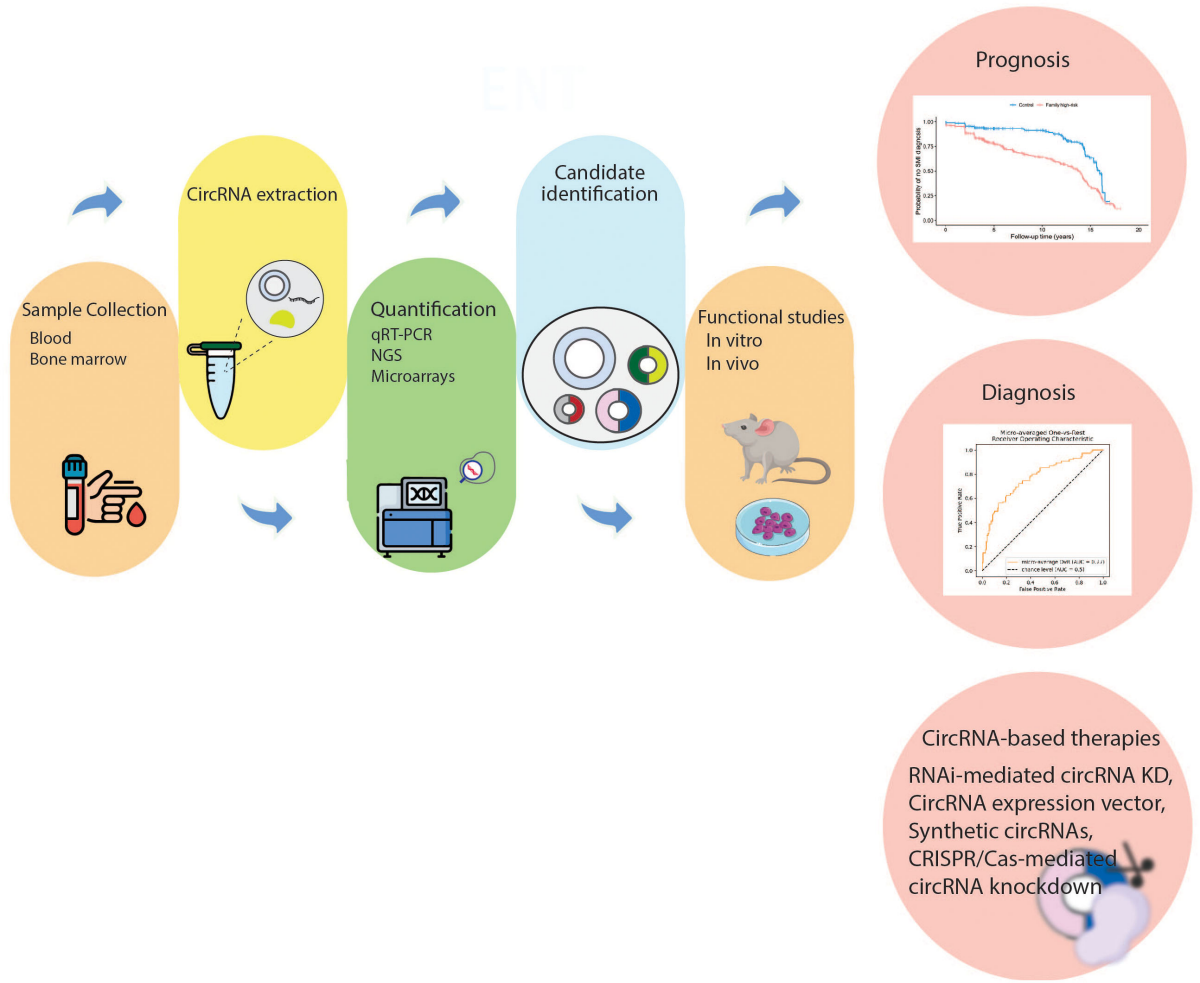
Clinical relevance of circRNAs in AML. The first step is sample collection (bone marrow, serum, or plasma) to be used for circRNA detection. Subsequently, dysregulated circRNAs are quantified through molecular techniques. Then, the mechanisms of dysregulated circRNAs in AML pathogenesis are investigated *in vivo* and *in vitro*. The dysregulated circRNAs that can act as diagnostic or prognostic biomarkers are selected. Moreover, targeting specific circRNAs may be an attractive treatment strategy. CircRNA, circular RNA; NGS, next generation sequencing; qRT-PCR, real-time quantitative polymerase chain reaction; RNAi, RNA interference; KD, knockdown.

The role of circulating blood components, such as circulating tumor DNA (ctDNAs), exosomes, circulating tumor cells (CTCs) and miRNAs for diagnostic and therapeutic applications has been gradually revealed (187–189). CircRNAs can have early diagnostic and high prognostic value in AML due to their unique structure, which is not easily degraded by RNA hydrolases and has a high stability in body fluids such as blood and urine. Therefore, they can be used as new non-invasive diagnostic methods (190, 191). In addition to the biological function of circRNAs in leukemia development and initiation, the identification and targeting of these abundant and stable molecules may have a variety of practical uses in the clinical management of AML. These include acting as markers for disease diagnosis and classification, serving as biomarkers of prognosis to help direct treatment decisions, and possibly could be used as promising targets for drug treatment (**Figure 3**). Next, we will briefly discuss the relationship between several circRNAs and their clinical valuable of AML. Several important diagnostic and therapeutic circRNAs are summarized in **Table 2**.

**Table 2:** Summary of candidate circRNAs that could be diagnostic and prognostic biomarkers of AML.

CircRNAs	Expression	Specimen	Sample size	Diagnostic value	Kaplan-Meier analysis	Ref.
4-circRNA signature (circKLHL8, circFCHO2, circSMC1A, circCFLAR)	Down-regulated	BM, blood	365	NR	↑* These circRNAs positively associated with DFS, OS, and EFS	(57)
RNF220	Up-regulated	BM, blood	87	AUC value _BM/PB_: 0.926/0.960 Sensitivity value _BM/PB_: 85.25%/90.48% Specificity value _BM/PB_: 93.98%/97.06%	↑ CircRNF220 at the time of diagnosis correlated with a low RFS rate	(66)
ANAPC7	Up-regulated	BM	144	AUC value: 0.915	No influence on the OS and DFS	(151)
circ-0009910	Up-regulated	BM	70	NR	↑ Circ_0009910 associated with shorter OS	(70)
circ-0009910	Up-regulated	BM	37	AUC value: 0.916 Sensitivity value: 81.08% Specificity value: 86.49%	↑ Circ_0009910 correlated with worse OS and prognosis	(72)
circ_vimentin (circ_VIM)	Up-regulated	BM	113	AUC value _whole AML patients_: 0.741 AUC value _non-APL AML_: 0.740 AUC value _CN-AML_: 0.749	↑ Circ_vimentin associated with shorter OS and LFS.	(192)
hsa_circ_0006404 (circ_Foxo3)	Down-regulated	BM	122	AUC value: 0.633 Sensitivity value: 62.1% Specificity value: 75%	Patients with high circFoxo3 survived longer	(193)
circ-ANXA2	Up-regulated	BM	130	AUC value: 0.832	↑ Circ-ANXA2 correlated with shorter OS, EFS and CR and associated with poor-risk status	(85)
circPLXNB2	Up-regulated	BM	40	AUC value: 0.8525	↑ CircPLXNB2 correlated with shorter LFS and OS	(82)
hsa_circ_0004277	Down-regulated	BM	113	AUC value: 0.957	NR	(194)
hsa_circ_0012152	Up-regulated	BM	49	AUC value AML/NCs: 0.9773 Sensitivity value AML/NCs: 97.7% Specificity value AML/NCs: 100% AUC value AML/ALL: 0.8625 Sensitivity value AML/NCs: 77.5% Specificity value AML/NCs: 88.6%	NR	(86)

* ↑ Indicates high expression.

NR, not reported; AUC, Area under the ROC Curve; DFS, disease-free survival; OS, overall survival; EFS, event free survival; RFS, relapse free survival; LFS, leukemia free survival; CR, complete remission; NC, normal control; AML, acute myeloid leukemia; CN-AML, cytogenetically normal- acute myeloid leukemia; APL, acute promyelocytic leukemia; BM, bone marrow; PB, peripheral blood.

#### 3.2.1 CircKLHL8 and circFBXW7

AML patients that are classified as cytogenetically normal (CN-AML) make up most of all cases of AML, comprising 45%–60%. Due to normal cytogenetic features, the prognosis of CN-AML must be based solely on genetic mutations. Furthermore, clinical outcomes of patients in this subgroup vary widely and are difficult to determine (195, 196). Papaioannou et al. (57) developed a novel computational method named Massive Scan for circRNA (MScircRNA) to detect and quantify circRNAs of CN-AMLs. One hundred eighty circRNAs were robustly and differentially expressed among the CN-AML samples. These researchers identified four circRNAs, circSMC1A, circKLHL8, circCFLAR, and circFCHO2, as prognostic values. Patients with high expression of these circRNAs had higher OS rate, DFS, and event-free survival (EFS) than those with low expression. Univariate and multivariate Cox regression analyses discovered that 2 of these circRNAs, circKLHL8 and circFCHO2, were independent predictors of better clinical outcomes. In terms of biological roles in AML, circFBXW7 knockdown increased the proliferative ability of leukemia cells. There are no miRNA binding sites in circFBXW7, so miRNA sequestration is unlikely to explain these effects. Nevertheless, the expression pattern of circFBXW7 was associated with a distinct signature of the genes involved in the multiple key cellular pathways (57).

#### 3.2.2 Circ_vimentin (circ_VIM)

Circ_VIM expression has been shown to increase in AML tissues. Patients stratify into two groups based on circ_VIM expression pattern, circ_VIM high, and circ_VIM low. Aberrant expression of circ_VIM in AML tissues was correlated with WBC counts and FAB classification. However, no other significant relation with clinicopathological features including gender, hemoglobin, platelet and BM blasts, karyotype classification, and gene mutational status between the two groups was observed. Circ_VIM expression exhibited a diagnostic value with an AUC of 0.741, representing a potential diagnostic performance of circ_VIM for AML. Moreover, high expression of circ_VIM was correlated to poor prognosis for AML, with a low OS and leukemia free survival (LFS) (192).

#### 3.2.3 Hsa_circ_0006404 (Circ_Foxo3)

The human FOXO3 gene encodes circRNA forkhead box O3 (circ_Foxo). Recently, circ_Foxo3 has played a critical role in the pathogenesis of various cancers in a ceRNA manner (197). For example, circ_Foxo3 facilitate human glioblastoma progression through upregulation NFAT5 *via* targeting miR-138-5p/miR-432-5p (198). Circ_Foxo3/miR-143-3p/USP44 loop contributes to gastric cancer growth and progression (199). In contrast, circ_Foxo3 has tumor-suppressive roles in bladder cancer by up-regulating TGFBR2 *via* miR-9-5p (200). Zhou et al. (193) found that Foxo3 and circ_Foxo3 expression was decreased in AML clinical specimens and cell lines. By analyzing ROC, the accuracy of diagnostic value of Foxo3 and circ_Foxo3 had been assessed and AUC of 0.655 and 0.633 were seen, respectively. Based to Kaplan-Meier analysis in different classifications of AML patients, the group with high Foxo3 expression exhibited a more favorable prognosis than those with low expression of Foxo3. In the univariate and multivariate Cox regression test for assessment of prognostic factors, it was demonstrated that Foxo3 expression is a protective factor while age and karyotype classification are adverse predictors of prognosis in AML patients. Although Kaplan-Meier analysis showed that patients with high levels of circ_Foxo3 lived longer than those with low expression, no statistically significant correlation between circ_Foxo3 expression and OS time of AML patients was found. It is still possible that circ_Foxo3 will be valid for diagnosing and treating AML patients, despite the lack of identification of miRNAs associated with AML.

#### 3.2.4 CircPAN3

Recently, circRNAs have received much attention from various research groups for their role in drug resistance. CircRNAs are contributing to drug resistance mainly by their cellular functions such as cell cycle regulation, apoptosis, autophagy, intervention with the tumor microenvironment, dysfunction of DNA damage, alteration in drug targets, and regulation of drug efflux transporters (167). Shang et al. (83) investigated the patterns of circRNA’s expression in doxorubicin (ADM)-resistant THP1 and ADM-sensitive cells. 49 circRNAs had a differential expression pattern (35 of which were up-regulated, and 14 were downregulated) in ADM-resistant THP1 cells in comparison with naïve ones, suggesting circRNAs may be involved in the development of drug resistance in AML cells. Among the top ten up-regulated circRNAs, circPAN3 was selected for further analysis due to the potential association between its parent gene, PAN3, and leukemogenesis (201, 202). Overexpression of circPAN3 interferes with sensitivity of ADM, whereas its downregulation increases its sensitivity. These findings were also validated in clinical specimens, as AML patients with refractory/recurrent cancer expressed circPAN3 at significantly higher levels than chemo-sensitive patients. CircPAN3 functioned as miR-153-5p and miR-183-5p sponges and form the circPAN3/miR-153-5p-miR-183-5p/XIAP regulatory axis to induce drug resistance. Mechanistically, circPAN3 promoted X-linked inhibitor of apoptosis protein (XIAP) expression by direct interaction with miR-153-5p and miR-183-5p and inhibiting the expression of these two miRNAs. miR-183 was reported to be up-regulated in BM and serum of pediatric AML and function tumor-promoting role by regulating programmed cell death 6 (PDCD6) (203). MiR-153 expression was lower in melanoma tissues and cells than in normal adjacent tissue and melanocyte. Furthermore, miR-153 overexpression suppressed tumor cell proliferation and invasion but motivate cell apoptosis by targeting snail family transcriptional repressor 1 (SNAI1) (204). XIAP is a prominent protein member of the inhibitor of apoptosis (IAP) that can directly inhibit the activity of both initiation caspase and executioner caspase. Its overexpression leads to chemoresistance (205, 206). CircPAN3 also mediates drug resistance in AML through the regulation of autophagy. Furthermore, siRNA circPAN3 inhibition deregulated the expression of three autophagy markers, the ratio of LC3-II/LC3-I, Beclin-1, and P62 (84). Recently, it has been shown that autophagy underlies the resistance to various cytotoxic drugs in solid tumors (207, 208). Basal autophagic activity has been elevated in ADM-resistance cell lines and BM leukemic cells of the relapsed AML. Further analysis revealed that circPAN3 mediated autophagy activity through AMPK/mTOR activity.

#### 3.2.5 Circ-ANXA2

Ding et al. (85) using microarray analysis, found 354 aberrantly expressed circRNAs, including 173 up-regulated and 181 downregulated, in BM of AML patients compared with healthy controls. In GO and KEGG pathway enrichment analyses, dysregulated circRNAs were enriched in biological processes and several AML-related signaling pathways like the ErbB signaling, EGFR tyrosine kinase inhibitor resistance, and mTOR signaling pathway. Among ten selected candidate circRNAs, up-regulated circRNA annexin A2 (circ-ANXA2) seen to have the highest value in diagnosis with an AUC of 0.832, indicating that circ-ANXA2 may serve as a promising biomarker for detecting AML. More importantly, the OS and EFS, and CR for patients with high circ-ANXA2 level of expression were considerably shorter than for patients with low levels. Clinical studies revealed that high circ-ANXA2 expression was associated with higher disease risk, poor risk stratification, decreased CR, shorter EFS, and OS in AML patients. Furthermore, it has been proposed that circ-ANXA2 enhances the expression of its parental gene, ANXA2, which stimulates fibrinolysis and matrix invasion of leukemia cells and induce chemo refractoriness. However, ANXA2 gene expression was not evaluated in the study. Functionally, knockdown of circ-ANXA2 was observed to inhibit the proliferation and increase apoptosis and chemosensitivity to cytarabine and daunorubicin. In terms of mechanism, circ-ANXA2 could act as a sponge of miR-23a-5p and miR-503-3p, which were known to contribute to AML chemosensitivity and tumor growth inhibition *via* regulation of cancer stem cell proliferation and self-renewal (209, 210).

#### 3.2.6 Hsa_circ_0001257 (circ_PLXNB2)/hsa_circ_0004520

Extramedullary infiltration (EMI) refers to leukemic cells found in organs or tissue other than the blood or bone marrow. EMI can be found either at diagnosis or relapse, and even when patients receive a CR in the BM. Studies have indicated the poor prognosis of these patients with a lower OS and DFS (211). Several studies investigated the circRNA expression pattern in EMI to clarify the molecular mechanism underlying it and find biomarkers to help early diagnosis and treatment. In BM cells of EMI and non-EMI AML patients using whole-genome microarray and bioinformatics tools, Lv et al. (212) found that 548 circRNAs were differentially expressed with 253 up-regulated and 259 downregulated expressions. Among them, 17 circRNAs were further analyzed due to their key roles in critical regulating cell-cell cross-talk in EMI. 7 target genes of this up-regulated circRNAs, namely LRRK1, PLXNB2, OLFML2A, LYPD5, APOL3, ZNF511, and ASB2 were associated with poor prognosis, whereas two genes, PAPLN and NRXN3, indicated a more favorable prognosis. Additionally, the expression of hsa_circRNA_0004520 was evaluated due to its interesting target genes, PLXNB2 and VEGFA, which may participate in the angiogenesis pathway up-regulated in EMI AML BM samples.

More recently, it was noted that circ_PLXNB2 and its parent gene, PLXNB2, were increased considerably in patients with AML comparing to healthy control group. Notably, the level of circ_PLXNB2 was up-regulated even more in AML patients presenting with EMI than in patients with non-EMI AML. The expression of circ_PLXNB2 positively correlated with PLXNB2 mRNA expression. ROC analysis revealed the effective diagnostic performance of circ_PLXNB2 with an AUC of 0.8525 in distinguishing AML patients from healthy people. Moreover, OS and LFS indices were significantly shortened in patients with high level of expression of circ_PLXNB2 comparing to those with low expression. *In vitro* and *in vivo* circ_PLXNB2 knockdown inhibited cell growth and migration of leukemia cells and promoted apoptosis, while circ_PLXNB2 overexpression enhanced the characteristics mentioned above of AML cells (82).

#### 3.2.7 Circ-HIPK2

Li et al. (213) performed two independent ribosomal RNA-minus RNA-seq experiments on APL and identified 4313 circRNAs, including 1098 newly identified ones. An analysis of the dynamic expression of circRNAs during ATRA treatment was conducted by this research team on ATRA-treated cell lines, identifying 508 circRNAs that were up-regulated and 262 downregulated as a result of treatment. Additionally, most of these circRNAs undergo independent regulation from their parent linear mRNAs. Among them, circ-HIPK2, one of the downregulated circRNAs, considerably affected the ATRA-induced differentiation of APL cells. Mechanistically, circ-HIPK2 sponged miR-124-3p, which is a differentiation-associated miRNA. The circ-HIPK2 expression could be used as an APL biomarker, as APL patients expressed significantly lower levels of circ-HIPK2 than healthy controls and patients with other AML subtypes.

#### 3.2.8 Hsa_circ_0004277

Li et al. (194) found 147 up, and 317 downregulated circRNAs in AML patients with normal cytogenetic features with unique signatures varying based on AML risk status. Further, they identified a circRNAs-based molecular signature consisting of 5 abundant circRNAs, two up-regulated, hsa_circ_0035381 and hsa_circ_0049657, and three downregulated, hsa_circ_0001187, hsa_circ_0008078, and hsa_circ_0001947, which may help in patient risk stratification. Among them, a downregulated circRNA, hsa_circ_0004277, showed promising predictability in AML diagnosis, with an AUC of 0.957. Meanwhile, hsa_circ_000427 expression was significantly downregulated in a validation group of 107 AML patients at different phases of chemotherapy and was then returned to normal levels during remission and then regressed again at relapse. This study confirmed that the measurement of hsa_circ_0004277 on a serial basis might assist with surveillance and initial diagnoses of disease.

#### 3.2.9 Hsa_circ_0012152/hsa_circ_0001857

Guo et al. (86) conducted microarray analysis on patients with ALL and AML and identified ten differentially expressed circRNAs between the two groups. Among them, hsa_circ_0012152 was up-regulated in AML compared to ALL with an AUC of 0.8625. At the same time, the levels of hsa_circ_0001857 were significantly higher in ALL than in AML or healthy controls, with an AUC of 0.90911 indicating the discriminative potential of these two circRNAs. Specifically, the ceRNA network about hsa_circ_0012152 was also evaluated. Molecular mechanism research showed that hsa_circ_0012152 played an important role in leukemia initiation and progression through miR-491-5p/epidermal growth factor receptor (EGFR)/mitogen-activated protein kinase (MAPK1) or miR-512-3p/EGFR/MAPK1 regulatory axes. MAPK is a ubiquitous signaling molecule that regulates the growth, differentiation, and survival of many types of cells, including hematopoietic cells. Inappropriate MAPK activation may also play a role in the leukemic transformation of myeloid cells. It is repeatedly up-regulated in AML tissue and cells, thereby generating a poor prognosis for patients, specifically upon chemotherapy (214, 215). EGFR family belongs to type I receptor tyrosine kinases; its overexpression or mutation has been detected in solid tumors. Its expression was reported in about one-third of AML and is associated with poor clinical outcomes (216). Consistent with this report, hsa_circ_0012152 can regulate the proliferation of leukemia cells through the miR-625-5p/SOX12 axis. Hsa_circ_0012152 acts as a sponge of miR-625-5p, and, meanwhile, miR-625-5p, by downregulating SOX12, inhibits cell activity and induces cell apoptosis, thereby inhibiting AML progression. Notably, based on Kaplan-Meier analysis, the expression level of hsa_circ_0012152 was significantly correlated with OS: a low level of hsa_circ_0012152 presented a higher OS rate compared with patients with a high level of hsa_circ_0012152 (87).

#### 3.2.10 CircRNF220

In a study by Liu et al. (66) circRNF220 was identified as a stable and abundant circRNA in pediatric AML cells. Although RNF220 mRNA can give rise to 4 circRNAs, only circRNF220 was dramatically up-regulated in pediatric AML cells. It accurately discriminated AML from ALL and other hematological disorders, including myelodysplastic syndrome (MDS) and CML, with a sensitivity of 85.25%, a specificity of 93.98%, and an AUC of 0.926. Also, the values of sensitivity, specificity, and AUC were increased to 90.48%, 97.06%, and 0.9601, respectively, when the plasma level of circRNF220 was evaluated, Suggesting its potential application as a minimally invasive biomarker. Although circRNF220 expression did not associate with clinicopathological traits, it can play the role of an independent prognostic marker in early recurrence AML. Consistent with expectation, circRNF220 knockdown could suppress leukemia cells proliferation and induce apoptosis *via* binding to miR-30a and up-regulate its target genes MYSM1 and IER2. Several studies showed essential and cell-intrinsic functions of MYSM1 in HSCs and proposed that it might have a role in AML recurrence (73). While the role of IER2 in AML is not clear, it’s up-regulated levels in many human tumors have been reported, and it possesses an essential role in the regulation of tumor progression and metastasis (217).

## 4 Anti-oncogenic potential of CircRNAs and targeted therapy

As previously mentioned, circRNAs can serve as anti-oncogenes principally by up-regulating the tumor suppressor genes. Although these RNA molecules were primarily known to be ncRNAs, multiple studies have proven the presence of an open reading frame (ORF) that promotes ribosomal coding (218, 219). For instance, circ-ZEB1.19, circZEB-1.17, circZEB1.5, and circZEB1.33 trap miR-200 and are responsible for decelerating the lung cancer growth (220). The under-expression of circMTO1 was also seen in HCC, a circRNA that is identified to target miR-9, and thus promoting the expression of p21 tumor suppressor gene (221). The previously mentioned circRNAs have been suggested to involve in the suppression of lung cancer and HCC through sponging miR-9 (220–222). Interestingly, the artificially synthesized CSSD-9, containing multiple miR-9 binding sites, can also inhibit lung tumor progression and metastasis (222). Furthermore, a circular transcript from the SNF2 histone linker PHD RING helicase (SHPRH) gene, called circSHPRH, can protect a specific downstream protein from proteasomal degradation, and subsequently exerts its suppressor activity against glioma cells; the corresponding protein (i.e., SHPRH) will ubiquitinate PCNA antigen and inhibits cell proliferation (175). Profiling circRNAs’ expression patterns in CN-AMLs showed that circKHLH8 was associated with thrombocytosis, as well as a decrease in the number of blasts in peripheral blood; a finding that was correlated with improved survival in AML patients. Further, circFBXW7 down-regulation facilitated the progression of AML (223). Generally, through inhibiting miRNAs or targeting transcription factors towards tumor suppressor genes, circRNAs may act as anti-oncogens to enhance the expression of tumor-suppressor genes.

CircRNA-miRNA-mRNA axes are proven central networks, contributing to the onset and progression of leukemia, and therefore targeting these molecular interactions can lead to the development of potential therapeutic strategies. There is limited data about circRNAs’ contribution to the leukemia therapy; however, as a single circRNA with several binding sites for a vast array of miRNAs, suppressing the axis at its first step (i.e., the inhibition of circRNA expression) is more reliable than silencing a single miRNA or gene. Although circRNA inhibition results in maintaining the corresponding anti-oncogenic miRNA against XIAP, Rab10, PRACKB, or TLR pathway (38, 39, 55, 224), it is difficult to eliminate circRNAs without altering the existing genes. To specifically inhibit a circRNA rather than linear RNAs, the guide sequence should be directed at a circRNA’s distinct site that is called a back-splice junction (BSJ). CircRNA is not generally a protein-coding molecule, and thus its insertions/deletions (INDELs)-mediated depletion is burdensome. By substituting the circRNA-related gene with a marker gene, CRISPR-Cas-assisted homologous recombination may help conquer the aforementioned challenge (225), as well as avoiding undesirable mutations (226). Nevertheless, a mammalian circRNA locus (Cdr1) was shown to be successfully depleted from the mice genome by CRISPR-Cas9 technology (227). Also, the interference with RNA pairing process attenuates the circRNA expression, leading to the repression of a specific circRNA (e.g. circGCN1LI) in human PA1 cells (228). The recently established CRISPR-Cas13 approach, which employs a specific enzyme of type VI CRISPR-Cas system, strongly seems to be promising in differentiating linear transcripts and circRNAs relying on its specific affinity to the BSJ site (229). High-throughput screening can also be performed with CRISPR-Cas-based circRNA engineering tools (230). Researchers may attempt to facilitate global screening with the use of well-characterized guide RNA (gRNA) libraries designed for circRNAs in the future.

## 5 Perspectives and challenges

There are tremendous issues in the field of circRNAs-leukemia interactions. As an example, there is no gold-standard tool to compare data, and highly-priced NGS technologies have made researchers to decrease the sample size. Even though research has conducted on circRNA biogenesis, little is known about how they are processed inside the cells. The expansion of circRNAs can be cytotoxic due to their high stability, and evidence suggests that excessive circRNAs can be exported by exosomes (231). Therefore, much molecular-based research should be performed on circRNA metabolism *in-vivo*. Another challenge should be addressed through further studies, which could name circRNAs concerning their host genes along with the term “*circ*”. Additionally, unreliable web-based tools have represented low agreement between circRNAs that have been tested virtually. Future evaluations should be conducted on molecular-based mechanistic and functional database to direct the research.

Our knowledge of circRNAs’ role in AML is limited; it has been suggested that miRNA sponging is an effective process in AML, and further focus on other mechanisms, such as circRNA-RBP or circRNA-DNA interactions, is needed. Analysis of the obtained data demonstrates that deregulation of circRNAs, in almost all cancer types, causes the transcription, miRNA sponging, protein translation, and protein sponging being deregulated. Since genetic and epigenetic alterations are central to the development of cancer cells, it should be considered that there is an association between the circRNAs deregulation and the corresponding changes. Recently, mechanisms involved in circRNA deregulation have been investigated to be contributed to a wide variety of malignancies. More investigations and further characterization should be designed and developed to reveal the exact function of these circular structures in different cancers, especially AML, and as the result, to identify emerging biomarkers with diagnostic, prognostic and even therapeutic potentials in AML.

## 6 Conclusion

Although formerly circRNAs were known as noise during the RNA splicing process, the development of RNA-sequencing technologies has led to the improvement of our knowledge about the functions of this amazing group of ncRNAs. The substantial roles of circRNA in tumorigenesis has been determined by the observed alterations in their expression levels. Once these RNA molecules interact with miRNAs or proteins, prominent regulatory networks are formed in the process of cancer progression. Also, the accumulated evidence has shown considerable diagnostic and therapeutic capacities for circRNAs in multiple cancers. The role of circRNAs in AML progression have been studied at different molecular mechanisms. Given the interactions between circRNAs and DNA/proteins, it can be concluded that these amazing molecules may be considered as promising candidates for both diagnosis and treatment of AML.

## Author contributions

AR and AM: Contributed in conceptualization, design, writing, and draft manuscript preparation; FS, ZBA, NM, BG, RA, MA, OV, MH, ZA, MS, AA, HM, EA: Contributed in reviewing relevant literature and writing; HM and EA: Supervision, review, editing and Validation. All authors read and approved the final manuscript.
